# Optical birefringence imaging of x-ray excited lithium tantalate

**DOI:** 10.1063/1.4997414

**Published:** 2017-08-01

**Authors:** S. M. Durbin, A. Landcastle, A. DiChiara, Haidan Wen, D. Walko, B. Adams

**Affiliations:** 1 Department of Physics and Astronomy, Purdue University, West Lafayette, Indiana 47906, USA; 2 Advanced Photon Source, Argonne National Laboratory, Argonne, Illinois 60439, USA

## Abstract

X-ray absorption in lithium tantalate induces large, long-lived (∼10^−5^ s) optical birefringence, visualized via scanning optical polarimetry. Similar birefringence measured from glass, sapphire, and quartz was two orders of magnitude weaker; much of this reduction can be accounted for by their smaller cross section for x-ray absorption. While x-ray induced charges can perturb local refractive indices and lead to birefringence, aligned dipoles in the non-centrosymmetric unit cell of ferroelectric LiTaO_3_ create electric fields that also induce birefringence via electro-optic coupling, which shows up as a dependence on crystal orientation. Time-resolved measurements from LiTaO_3_ show a prompt response on a picosecond time scale, which along with the long decay time suggest novel opportunities for optical detection of x-rays.

Lithium tantalate (LiTaO_3_) is an electro-optic material widely used to control, modify, and detect light through large refraction changes induced by electric fields. Ultrafast induced birefringence, for example, allows temporal profiles of electric pulses in LiTaO_3_ to be measured with sub-picosecond precision.^1,2^ The electro-optic response is also responsible for the photorefractive effect, where a pump light pulse induces refractive changes that can then be sensed by a probe pulse.^3–5^ In one example involving holographic storage in Fe-doped LiTaO_3_, it was shown that prior electronic excitation by UV light strongly enhances the photorefractive signal written by an IR pump pulse, indicating the complex role of defects in this ferroelectric material.^6^ In this letter we report on dramatic refractive effects in LiTaO_3_ created by intense synchrotron x-ray pulses. Scanning optical birefringence microscopy reveals x-ray induced electric field strengths approaching 10^6^ V/m, prompt initial response times at the picosecond level or faster, and non-exponential decay times extending beyond 10 *μ*s. We also report observations of x-ray excited acoustic waves and strongly perturbed optical reflectivity. These effects are attributed in part to the formation of aligned electric dipoles within the non-centrosymmetric unit cell (trigonal crystal system, space group R3c)^7^ and suggest new possibilities for polarization imaging for ultrafast x-ray detection.

The optical arrangement is briefly sketched in Fig. 1. Laser pulses at 780 nm, <50 fs duration, and 88 MHz repetition rate were focused onto the lithium tantalate sample surface with a 5× microscope objective, yielding a spot size of ∼5 *μ*m and an average laser power around 5 mW. Transmitted light was recollimated by a second lens, separated by a polarizing beam splitter into parallel and perpendicular components, and detected by a photodiode. The laser beam was linearly polarized and oriented to produce approximately equal parallel and perpendicular intensities after the beam splitter. Optical grade lithium tantalate wafers with 0.5 mm thickness and the c-axis oriented in the surface plane (“X-cut”) were acquired commercially and cut into smaller rectangles.^8^ This material is not intentionally doped, has a quoted material purity over 99.995%, and is the standard congruent (non-stoichiometric) composition where several percentages of Li sites are vacant or contain Ta antisite ions.^9,10^

**FIG. 1.**
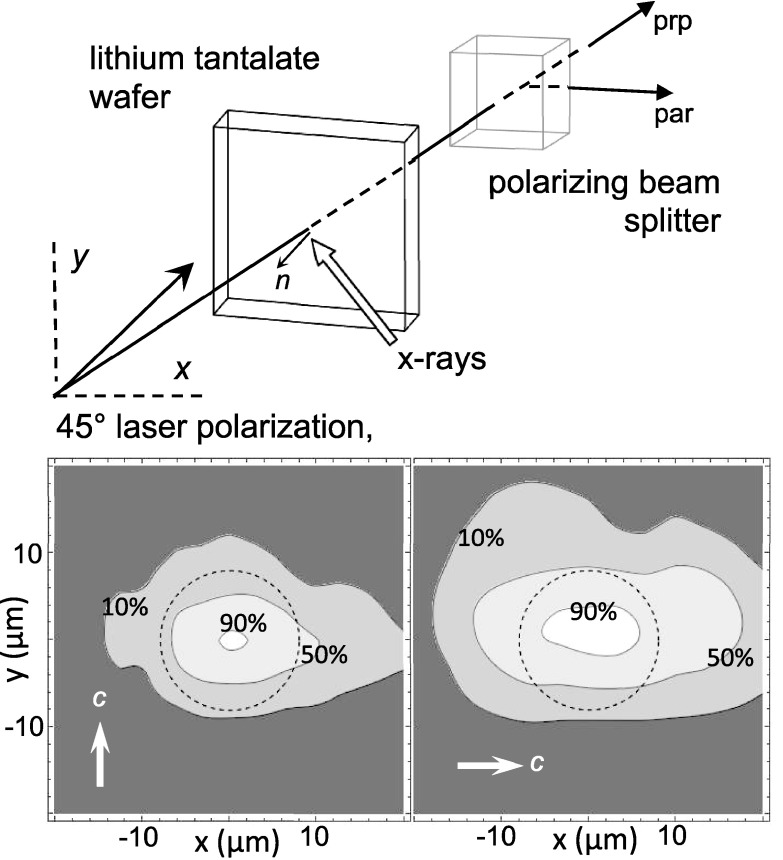
Detection of x-ray induced optical birefringence in lithium tantalate. Top: a 45° linearly polarized pulsed laser beam is transmitted through a lithium tantalate wafer and split into orthogonal components by a polarizing beam splitter cube; not shown are focusing and recollimating lenses before and after the sample. Intensities are measured as the laser is rastered across the x-ray target spot. Laser and x-ray beams are in the horizontal plane, and the x-rays are horizontally polarized. Bottom: contour plots of the birefringence signal for vertical and horizontal orientations of the lithium tantalate optical axis. X-ray beam size (16 *μ*m FWHM) is indicated by the dashed circles.

Measurements at the Sector 7 insertion device beamline of the Advanced Photon Source synchrotron^11^ used monochromatic 12-keV x-rays with 60-ps (FWHM) pulses at 88 MHz, synchronized with the laser pulses, that were focused to a 50-*μ*m spot size with x-ray Kirkpatrick-Baez mirrors and further reduced to a 15-*μ*m diameter with slits. At this x-ray energy, the penetration depth in lithium tantalate is 7.7 *μ*m.^12^ The incident flux was 10^3^ x-ray photons (2 × 10^−12^ J) per pulse, for an average power of ∼0.2 mW. An optical chopper modulated the x-ray beam at 500 Hz. These x-rays are over 90% linearly polarized in the horizontal direction.

The laser and x-ray beams were directed in a horizontal plane to the vertical sample surface, each symmetrically at 30° away from the surface normal (Fig. 1). The optical components were mounted on a motorized stage for scanning across the sample surface and fixed x-ray spot. The outputs of the two photodiode detectors were connected to differential inputs of a lock-in amplifier referenced to the x-ray chopper frequency, ensuring that the output signal registered only the change in birefringence caused by the x-rays.

The birefringence signal depends primarily on the component of the electric field in the c-axis direction, integrated along the transmitted beam path.^13^ To determine the electric field strength versus birefringence, we installed a lithium tantalate specimen with top surface gold strip lines perpendicular to the in-plane optical axis.^1^ The laser was focused in the 30 *μ*m strip line gap, and the birefringence signal measured for gap voltages up to 10 V, or 3.3 × 10^5^ V/m, yielding a linear response. The strip line gap width is similar to the x-ray beam size, and the field penetration depth is comparable to the x-ray absorption length, so this provides a reasonable calibration of the x-ray induced birefringence versus local electric field.

The scanning transmission polarimetry images shown in Fig. 1 plot the magnitude of the x-ray induced birefringence as the laser spot is raster scanned in 3-*μ*m steps across the lithium tantalate surface, with clear differences between vertical and horizontal orientations of the optical axis. Using the calibration described above, the peak x-ray induced electric field is ∼7 × 10^5^ V/m, which using the electro-optic coefficients for lithium tantalate^13^ corresponds to an index of refraction change of ∼10^−4^. (Note that electric field strengths orders of magnitude greater have been reported for synchrotron x-ray absorption studies of frozen protein crystals.^14^)

The dominant absorption mechanism for 12-keV x-rays in lithium tantalate is the photoexcitation of *L* electrons in Ta. Photoelectrons and subsequent Auger electrons lose energy primarily through inelastic scattering from valence electrons.^15^ Induced electric fields in a ferroelectric material are produced by aligned dipoles, but the actual unit-cell distribution of x-ray generated electrons and holes in lithium tantalate is not known. (We note that a physical defect model of lithium vacancies and tantalum antisites has been proposed and may prove to be relevant here also.^16^) The optic axis is the direction of the intrinsic polarization in lithium tantalate, so we assume that is the orientation of x-ray induced dipoles.

To test the role of ferroelectricity, we repeated these measurements with non-ferroelectric quartz, sapphire, and ordinary glass specimens. Quartz, like lithium tantalate, has a non-centrosymmetric unit cell and is optically active. Sapphire is centrosymmetric and optically active, and glass (a microscope cover slip) is non-crystalline and optically isotropic. These data reveal that the lithium tantalate response is about a hundred times larger than the others. Much of this enhancement, however, is due to stronger x-ray absorption which concentrates the excitations closer to the surface; the x-ray absorption lengths are 7.7 *μ*m for LiTaO_3_ *vs*. 274 *μ*m for sapphire, 352 *μ*m for glass, and 359 *μ*m for quartz.^12^ Figure 2 displays the peak birefringence scans of these materials scaled by their relative absorption lengths. “Ordinary” birefringence results from x-ray induced charges locally perturbing the refractive indices. The normalized scans show that this effect can be a significant fraction of the lithium tantalate response. The large electro-optic coupling associated with ferroelectric ordering apparently increases the birefringence and causes the crystal orientation dependence shown in Fig. 1.

**FIG. 2.**
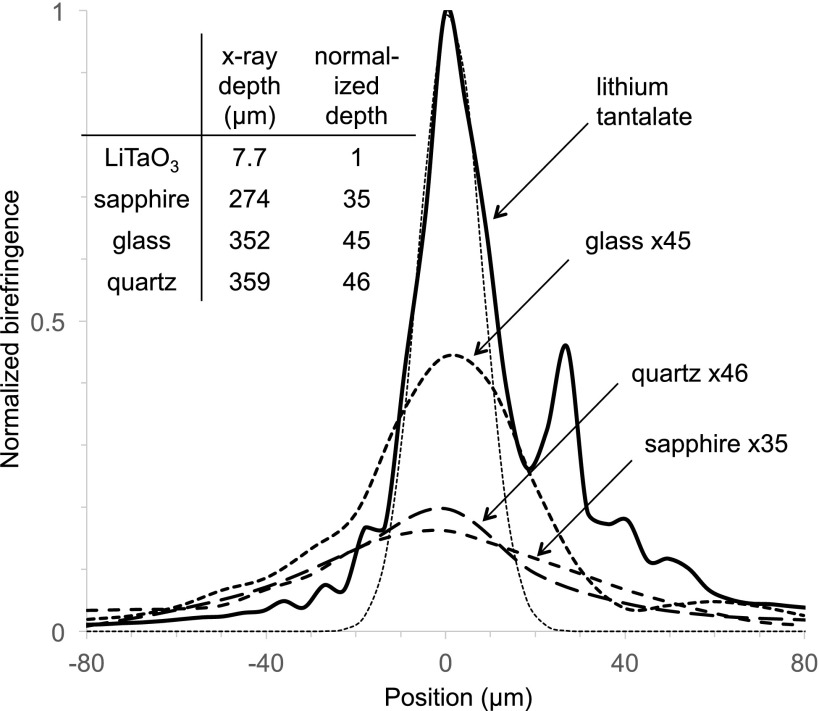
Normalized comparison of x-ray induced optical birefringence in lithium tantalate with quartz, sapphire, and glass. Scans are multiplied by the x-ray penetration depth, relative to lithium tantalate. Data obtained with x-ray beam width of 35 *μ*m; inner dashed curve shows a 16 *μ*m Gaussian fit to lithium tantalate. Inset: Table of x-ray penetration depths, absolute and normalized to lithium tantalate.

The temporal response was investigated at APS Sector 14, which was configured to provide 500-Hz x-ray pulses of ∼90-ps (FWHM) duration with over 90% horizontal polarization.^17^ The 12 keV x-rays had a pulse energy of ∼15 *μ*J, an enormous enhancement due to using the full bandwidth of specially designed dual undulators. The x-ray beam spot was focused to 50 *μ*m, with the laser focused to half this size. The laser had 780-nm pulses of 3 ps (FWHM) duration with a 1-kHz repetition rate, synchronized so that every other laser pulse followed a 90-ps x-ray pulse with a chosen time delay; the lock-in amplifier was referenced to the 500-Hz x-ray frequency. The horizontal x-ray beam intersected the vertical laser beam at the sample surface, oriented with its surface normal in the plane of the x-ray and laser beams and rotated 30° away from the laser (see Fig. 3, lower inset). The laser polarization analysis was the same as before (Fig. 1), with the input laser beam linearly polarized and adjusted to approximately null the difference in perpendicular and parallel output signals at the lock-in.

**FIG. 3.**
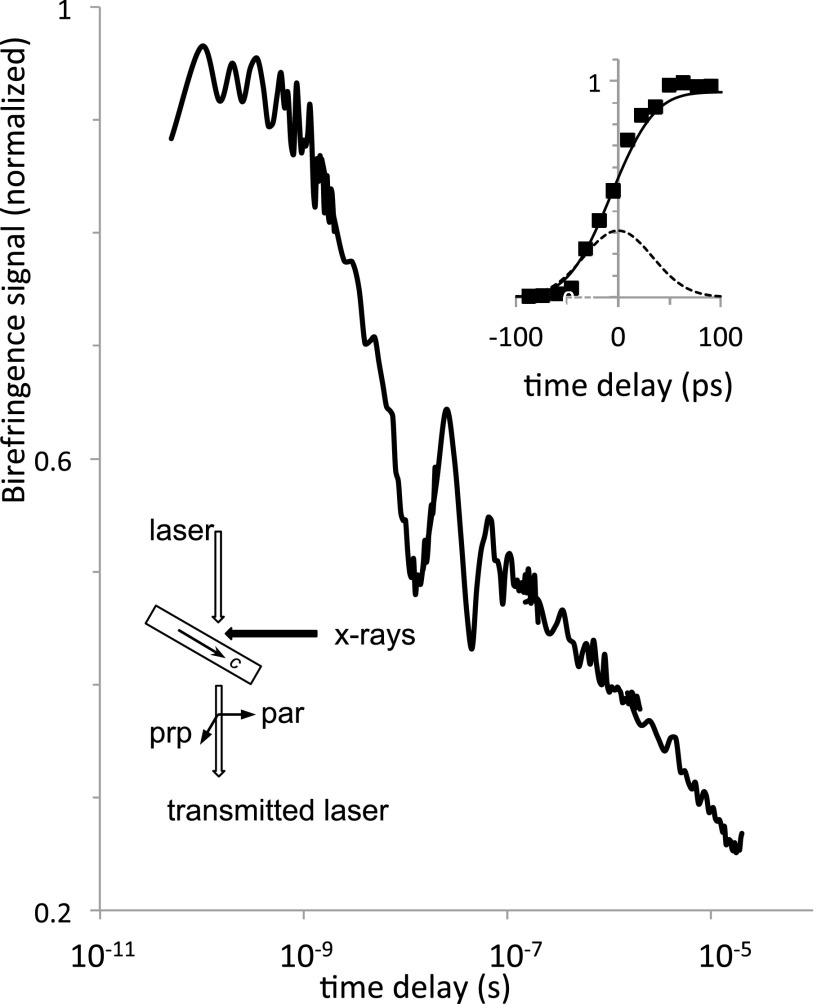
Time response of x-ray induced birefringence in lithium tantalate. Using 90 ps x-ray pulses, birefringence exhibits a non-exponential decay; the oscillation near 10^−8^ s is attributed to a bulk acoustic wave. Upper inset: initial response compared to the x-ray pulse profile (dashed line) and its integral (solid line). Lower inset: side-view of laser and x-ray beams at the sample, with c-axis denoted by arrow. X-rays are polarized horizontally (out of the page), and the linear polarization direction of the laser bisects the orthogonal “prp” and “par” directions.

The early-time data (Fig. 3, upper inset) reveal that x-ray induced birefringence, and hence the underlying charge distribution responsible for the strong electric fields, turns on at a rate indistinguishable from the integrated x-ray flux. That is, any time delay between the absorption of x-rays and the creation of the electric field cannot be longer than a few picoseconds and potentially is much faster. Furthermore the signal persists beyond 1 *μ*s, with a slow, non-exponential decay (Fig. 3) indicative of a complex set of energy levels that govern recombination. (This is similar to the time scale for UV erasure of photorefractive signals in Fe-doped lithium tantalate.^6^) The prompt turn-on could make lithium tantalate useful as an optical ultrafast x-ray detector material, where the birefringence signal could track integrated x-ray flux at synchrotrons or x-ray free electron laser (XFEL) sources. The relatively slow decay is well matched to low repetition rate XFELs but might be a poor match for synchrotron sources where pulse intervals are typically much shorter than microseconds.

Lithium tantalate was further characterized by measuring laser reflectivity induced by x-ray absorption (Fig. 4), which showed a time-dependent relaxation quite similar to the transmission birefringence (Fig. 3). The peak x-ray induced change in laser reflectivity is nearly 15% of the incident light. The prominent oscillation at *Δt* ∼ 3 × 10^−8^ s apparently arises from a bulk acoustic wave excited by the x-ray pulse. Assuming this pulse travels at the speed of sound (*v*_*s*_ = 7 × 10^3^ m/s^18^), the approximate depth is *Δx* = *v*_*s*_ *Δt* ∼ 200 *μ*m. Further characterization will be required to establish the details of this bulk lattice excitation.

**FIG. 4.**
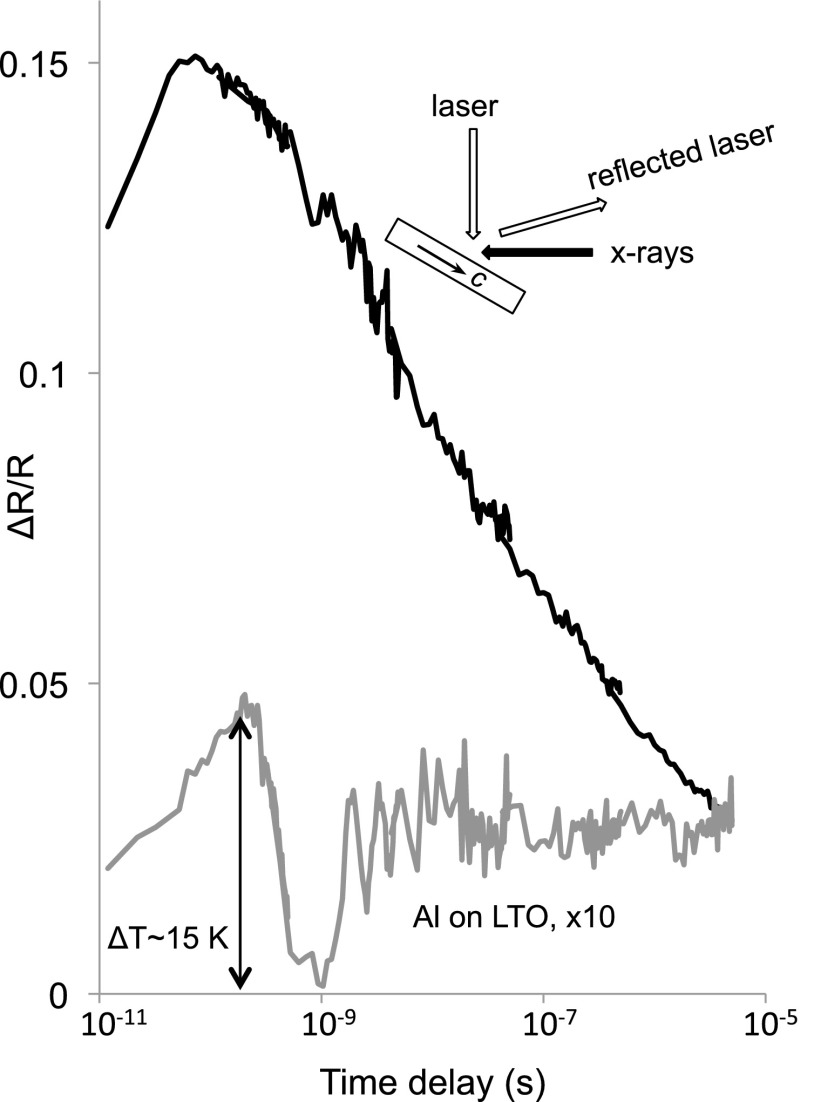
Time response of x-ray induced optical reflectivity from lithium tantalate (upper curve) and from Al-coated lithium tantalate (scaled by ×10), indicating a maximum temperature increase of 15 K from the intense x-ray pulses; the dip at 10^−9^ s is attributed to an acoustic wave excited by the x-ray pulse.

Because of the high x-ray pulse energy density (15 *μ*J in a 50 *μ*m spot) raises concerns about local heating, the resultant temperature rise was directly measured with thermoreflectance from a 30 nm aluminum film deposited onto a lithium tantalate specimen.^19^ Figure 4 shows the x-ray induced optical reflectivity from aluminum was over 30 times weaker than from bare lithium tantalate and corresponds to an initial temperature rise of only 15 K,^20^ which is unlikely to account for the strong birefringence and reflectivity responses.

This thermoreflectance technique is based on the optical reflectivity of aluminum smoothly changing as it expands with increasing temperature. These data show an initial rise and then a dramatic drop in reflectivity around 1 ns, however, which certainly cannot be explained as a real drop in temperature. Instead we attribute this rise and fall to the same acoustic wave pulse seen in the birefringence data (Fig. 3). The reflectivity drop is simply due to lattice contraction, part of the initial acoustic wave pulse excited by absorption of the x-ray pulse. Optical reflectivity, being surface sensitive, would detect the initial (∼10^−9^ s) acoustic response to the x-ray impulse, while birefringence is measured in transmission through the bulk and sees the largest response later (∼3×10^−8^ s) after propagation nearly midway through the sample.

We explored the role of just the aligned dipoles with a simple model that assumed the absorbed x-ray energy is converted into dipoles with 50% efficiency, in which we associate each dipole with a separation of 0.2 nm aligned with the c axis and a formation energy of 4.0 eV. The x-ray beam profile and absorption length then gives the dipole distribution $\vec{P}\left(\vec{r}\right)$ (Fig. 5, top panel). The induced charge density $\rho =-\nabla \cdot \vec{P}$ for the two different crystal orientations was then calculated (Fig. 5, center panels), the electric potential was determined from Poisson’s equation, and the electric field was determined from $\vec{E}=-\nabla \cdot \vec{V}$. Rotation of the laser polarization, and hence the birefringence, is proportional to the local component of the electric field parallel to the optic axis, integrated along the laser path length.^2^ This is calculated as a function of position transverse to the laser beam, and the resulting scans are shown in Fig. 5 (bottom panels) for both orientations.

**FIG. 5.**
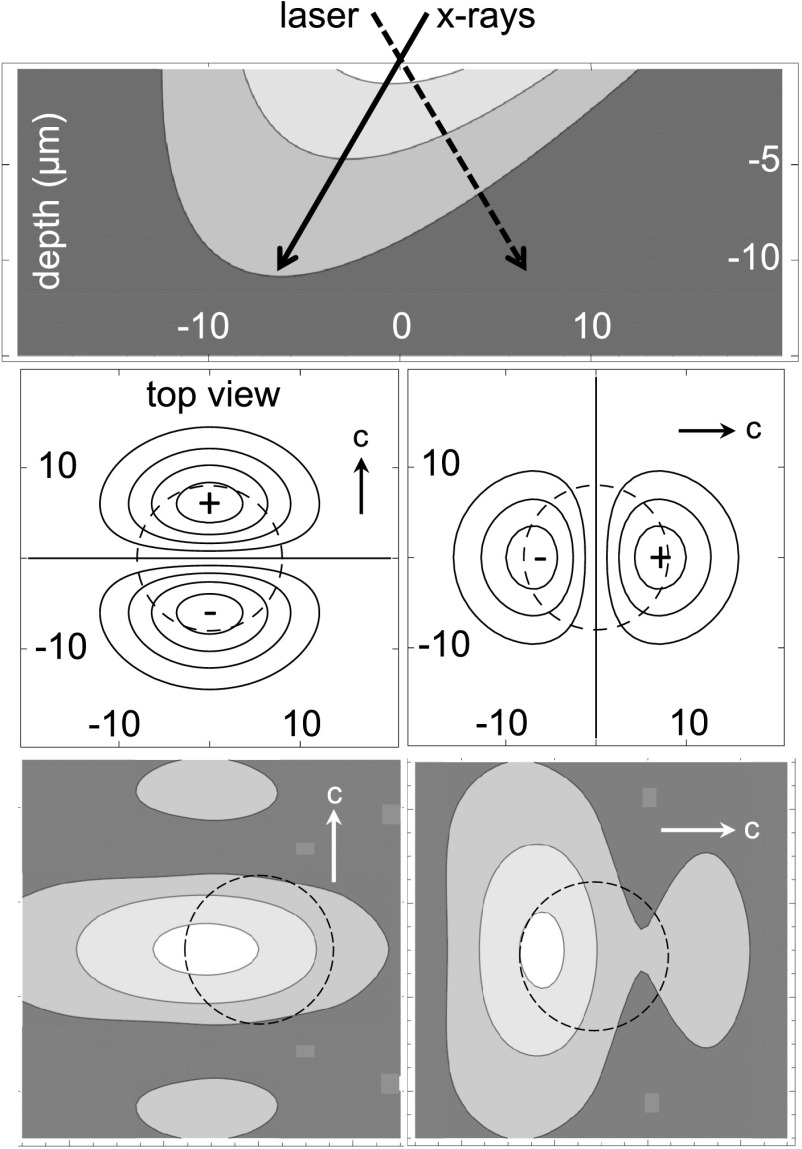
Simulation of x-ray induced birefringence in lithium tantalate. Top: cross section view of the specimen, showing the incident x-ray and laser beams and the induced dipole distribution as a function of depth. Scale numbers are in microns, and contour levels are 90%, 50%, and 10% of the maximum dipole density. Center: corresponding surface charge densities (contour levels in 20% increments) for the optical axis perpendicular (left panel) and parallel (right panel) to the plane of the x-ray and laser beams. Dashed circles are the 16 *μ*m x-ray beam footprint. Bottom: electric field components in the c-axis direction sensed by the laser beam along its path through the sample (90%, 50%, and 20% contour levels). Dashed circles are the x-ray beam footprints.

While these simulated results do confirm that the scan profiles depend on the c-axis orientation, they do not reproduce some of the main features seen in Fig. 1. This suggests that the ordinary birefringence exhibited by glass, for example, also makes a significant contribution in lithium tantalate. We also note first that the simulation does not consider time-dependent migration of charges over the ∼10^−5^ s recombination time. Mean free paths greater than 10 *μ*m were reported for x-ray excited free carriers in doped lithium niobate;^21^ given electric fields exceeding 10^5^ V/m, electrodiffusion of charge is likely to smooth certain features of the simulation. We conclude that aligned dipoles associated with ferroelectric symmetry are necessary to account for the dependence on crystal orientation, but ordinary birefringence is also important. Further studies will be required to determine the complete mechanism for the x-ray induced birefringence, including the details of charge distribution and the occupied sites within the non-centrosymmetric unit cell.

Lithium tantalate’s picosecond response for hard x-ray induced optical birefringence and reflectivity suggests applications as an optical time-resolved x-ray detector. Currently there are no practical picosecond electronic detectors for x-rays,^22^ but several studies have shown fast optical responses for GaAs and silicon nitride, with some application at free electron laser sources.^23–26^ Lithium tantalate offers the possibility of stronger response to hard x-rays, plus a birefringence transmission geometry insensitive to detector thickness. Significant enhancements may be obtained using materials with larger electro-optic coefficients, smaller band gaps, and faster recovery times for synchrotron applications.

To summarize, the absorption of intense x-ray beams in lithium tantalate induces large internal electric fields and greatly enhanced optical birefringence. The turn-on of this effect tracks the temporal profile of the absorbed x-rays with picosecond fidelity (or better) but decays non-exponentially over tens of microseconds. This suggests possible applications as an optical time-resolved x-ray detector well suited for x-ray free electron lasers. Materials with faster decay times would be required for high repetition rate synchrotron x-ray sources.
